# Protein embeddings reveal a continuous molecular landscape of host adaptation in waterfowl parvoviruses

**DOI:** 10.3389/fbinf.2025.1738737

**Published:** 2026-01-27

**Authors:** Nihui Shao, Yunfei Guo

**Affiliations:** 1 Faculty of Science, University of Bern, Bern, Switzerland; 2 Animal Infectious Disease Laboratory, School of Veterinary Medicine, Yangzhou University, Yangzhou, China

**Keywords:** host adaptation, molecular continuum, protein language model, structural–electrostatic remodeling, VP1 protein, waterfowl parvovirus

## Abstract

Viral adaptation across closely related hosts often proceeds through subtle molecular changes that escape detection by classical phylogenetic analyses. In waterfowl parvoviruses, we integrate AI-based protein language modeling, structural biophysics, and infection assays to reveal a continuous trajectory of host adaptation linking Goose parvovirus (GPV) and Muscovy duck parvovirus (MDPV). Protein embeddings of VP1 sequences reveal a smooth manifold bridging GPV and MDPV, which softens an apparent phylogenetic dichotomy into a graded molecular topology. Structural modeling identifies a flexible surface loop (residues 300–420) as a biophysical pivot. Along the embedding trajectory, this loop undergoes gradual conformational expansion and electrostatic neutralization, quantitatively linking embedding coordinates to capsid surface remodeling. Experimentally, a GPV-type isolate recovered from naturally diseased ducks replicated efficiently in duck embryos, duck embryo fibroblasts, and live ducklings, producing characteristic lesions. These results show that waterfowl parvoviruses evolve along a continuous molecular–electrostatic landscape in which cumulative structural adjustments enable cross-host infectivity. Our framework connects AI-derived molecular representations to biophysical mechanisms and biological function, supporting a model of viral host adaptation as a predominantly continuous process and providing a foundation for predicting cross-host potential in emerging viral systems.

## 1 Introduction

The stability and gradual drift of viral host spectra are central to understanding how pathogens cross species barriers and adapt to new hosts (Longdon et al., 2014; Parrish et al., 2008; Woolhouse et al., 2005; Geoghe et al., 2018). Host-range changes rarely occur as single, isolated events; rather, they arise from continuous molecular processes driven by receptor recognition, structural remodeling, and immune evasion. Capturing these subtle but cumulative shifts at the molecular level helps explain how viruses breach host boundaries and establish new adaptive lineages, forming a framework for understanding zoonotic potential. Yet, when host species are phylogenetically close, these molecular drifts become entangled within minimal sequence divergence, creating an evolutionary “gray zone” that escapes resolution by traditional phylogenetic trees and masks fine-scale adaptive transitions.

Classical phylogenetic reconstruction and multiple sequence alignment have long been the foundation for studying viral host adaptation. Yet when sequence variation is low or host associations overlap, these methods frequently yield shallow branches or unresolved nodes that mask potential intermediate states in an evolutionary continuum. This limitation underscores the need for computational frameworks capable of capturing smooth molecular transitions and connecting classical phylogenetic relationships with the underlying biophysical dynamics of adaptation.

Waterfowl parvoviruses provide a suitable system for examining such continuous host adaptation. Goose parvovirus (GPV), a small single-stranded DNA virus belonging to the Parvoviridae family, has long been considered host-restricted to geese (Zádori et al., 1995; Tatár-kis et al., 2004). It causes Derzsy’s disease, a severe enteric condition marked by intestinal hemorrhage and necrosis, leading to significant economic losses in waterfowl production worldwide (Palya et al., 2009). A closely related lineage, Muscovy duck parvovirus (MDPV), primarily infects Muscovy ducks, and both were traditionally regarded as evolutionarily distinct (Zádori et al., 1995; Li et al., 2021). However, since the 2010s, GPV-like isolates have been detected in ducks in China and parts of Europe (Li et al., 2021; Ge et al., 2017; Chen et al., 2015). These isolates share over 95% nucleotide identity with classical GPV yet cause parvovirus-like disease in ducks, suggesting a molecular drift that bridges the GPV–MDPV host boundary.

Although our analyses focus on waterfowl parvoviruses, the underlying question is more general: how do structurally constrained, small, non-enveloped DNA viruses navigate host range evolution within the tight confines of compact capsids? Several families, including Parvoviridae, Circoviridae, and adeno-associated viruses, share a common architectural logic in which a conserved capsid core is decorated by surface-exposed loops that mediate receptor binding and immune recognition, and in which relatively modest changes in these loops can alter host range or tissue tropism without wholesale remodeling of the capsid scaffold (Mietzsch et al., 2019). Structural and functional studies in these systems have implicated gradual remodeling of surface loops as a plausible route to modulating receptor usage or host specificity, particularly in compact DNA viruses with highly constrained capsid architectures (Govindasamy et al., 2003). These observations suggest that continuum-like trajectories of host adaptation may recur within structurally constrained DNA virus families, even if they are not expected to extend to RNA viruses or large DNA viruses that evolve through more episodic, recombination-driven dynamics. Against this backdrop, waterfowl parvoviruses provide a tractable model for quantifying a host-associated continuum in a representative compact DNA virus lineage.

Recent developments in artificial intelligence have opened new possibilities for exploring such fine-scale evolutionary processes. Protein language models (PLMs), including ESM2, learn implicit evolutionary rules from large protein sequence datasets in an unsupervised manner, capturing continuous relationships among structure, function, and host specificity (Elnaggar et al., 2022; LaTourrette and Garcia-Ruiz, 2022; Min et al., 2016; Hie et al., 2021; Hopf et al., 2017). Unlike conventional phylogenetic trees that impose discrete branching, PLM-derived embedding spaces encode molecular similarity as continuous coordinates within a high-dimensional manifold, thereby reflecting gradual evolutionary transitions (LaTourrette and Garcia-Ruiz, 2022; Madani et al., 2023; Mao et al., 2019). This representation complements traditional approaches by characterizing host-associated sequence drift in geometric rather than temporal space. In parallel, advances in structural prediction—such as AlphaFold and RoseTTAFold—have demonstrated that these learned embeddings can be directly translated into accurate three-dimensional models, effectively linking statistical patterns to molecular structure (Baek et al., 2021; Townshend et al., 2021; Jumper et al., 2021).

Building upon these advances, we implemented an integrated framework combining AI-based prediction, experimental validation, and mechanistic interpretation (McInnes et al., 2018). Using the ESM2 model, we computed high-dimensional embeddings of the GPV VP1 protein and reconstructed host-associated topology through UMAP projection and k-nearest-neighbor classification (Cover and Hart, 1967; scikit-learn, 2024). The resulting embedding map revealed a continuous topology connecting goose- and duck-derived isolates, with several sequences occupying intermediate positions consistent with molecular transition states. Guided by this computational observation, we investigated a natural outbreak of duckling enteritis. RT-PCR and sequencing confirmed that the VP1 gene of the isolate clustered within the goose-origin GPV lineage, distinct from typical MDPV. Subsequent viral isolation and infection assays in ducklings provided biological validation for the AI-derived predictions. Collectively, these findings indicate that the GPV–MDPV system forms a continuous molecular and biophysical gradient rather than two discrete host-restricted lineages. The ESM2 embedding captures gradual electrostatic changes across key VP1 capsid regions, supporting the concept of “embedding drift”—a continuous, high-dimensional trajectory that reflects the molecular logic of viral host adaptation. This integrated framework, bridging AI embeddings, structural biophysics, and biological validation, provides a quantitative and interpretable approach to studying cross-host viral evolution.

## 2 Materials and methods

### 2.1 Research design and overall framework

This study used an integrated framework combining AI-driven molecular modeling, structural biophysics, and experimental validation to investigate continuous host adaptation in waterfowl parvoviruses. VP1 protein sequences from Goose parvovirus (GPV) and Muscovy duck parvovirus (MDPV) were obtained from public databases (Sayers et al., 2023). Phylogenetic reconstruction was first applied to establish lineage divergence, while AI-derived embeddings were used to capture continuous molecular transitions beyond tree-based classifications. Structural modeling and electrostatic analyses were performed to identify potential biophysical correlates of the embedding-derived continuum (Varadi et al., 2022; Dolinsky et al., 2004). Finally, both *in vitro* and *in vivo* infection assays were carried out to evaluate the replication capacity and pathogenicity of representative isolates (Xiao et al., 2017). All computational analyses were performed in controlled environments with fixed random seeds to ensure reproducibility, and experimental procedures followed institutional biosafety and animal ethics guidelines (National Academies Council, 2011).

### 2.2 Sequence collection and preprocessing

VP1, the major capsid protein of *Anseriform dependoparvoviruses*, was chosen as a molecular marker for host adaptation because of its key roles in receptor binding and immune recognition (Zádori et al., 1995; Tatár-kis et al., 2004; Li et al., 2021). All available GPV and MDPV VP1 protein sequences were downloaded from NCBI GenBank (as of December 2024) (Sayers et al., 2023). Sequences containing incomplete regions, artificial mutations, premature stop codons, or missing host metadata were excluded. Only full-length entries (700 ± 5 amino acids) were retained to ensure comparability across alignments. Metadata were standardized into four host categories: goose, duck, Muscovy duck, and unassigned. To minimize sampling bias, redundant sequences were removed, resulting in a curated dataset of 190 high-quality VP1 sequences representing diverse hosts and geographic origins.

### 2.3 Multiple sequence alignment and phylogenetic analysis

Sequences were aligned using MAFFT v7.505 with the L-INS-i algorithm (Katoh and Standley, 2013). Low-information or gap-rich regions were trimmed with trimAl v1.4.rev22 using the “–automated1” option (Capella-Gutiérrez et al., 2009). The resulting 703-site alignment was subjected to maximum-likelihood (ML) phylogenetic analysis in IQ-TREE v2.2.6, with ModelFinder used to determine the best-fit substitution model (Minh et al., 2020). Branch support was evaluated by SH-aLRT and ultrafast bootstrap tests (2,000 replicates each). Phylogenetic trees were visualized and color-coded by host category to provide a reference topology for the subsequent embedding analysis.

### 2.4 Protein language model embedding and host mapping

To characterize fine-scale molecular continuity, we used the Evolutionary Scale Modeling model (ESM2-T33_650M_UR50D) (Mietzsch et al., 2019) and cross-referenced related protein language models and design frameworks (Govindasamy et al., 2003; Elnaggar et al., 2022). Each VP1 sequence was encoded as a 1,280-dimensional vector by averaging per-residue embeddings. Dimensionality reduction was performed with UMAP v0.5.5 (parameters: *n_neighbors* = 15, *min_dist* = 0.1, *metric* = cosine) (McInnes et al., 2018) to visualize latent molecular topology.

Unlike conventional phylogenetic trees that impose discrete branching, AI-based embeddings represent molecular similarity in a continuous space, allowing the detection of gradual evolutionary transitions that may be unresolved by tree-based methods.

A 5-nearest-neighbor (kNN) classifier (Cover and Hart, 1967) was trained using embedding coordinates with known host labels to estimate host-association probabilities. Classification confidence was determined by the proportion of *kNN* votes. Embedding stability was assessed using the silhouette coefficient and neighborhood purity metrics (Rousseeuw, 1987), both of which indicated consistent manifold structure across random seeds. The global topology of the embedding remained stable under different parameter configurations, supporting its robustness.

### 2.5 Structural modeling and electrostatic potential analysis

Representative VP1 proteins from GPV, Transition, and MDPV lineages were modeled using the AlphaFold server (Varadi et al., 2022). For each lineage, we downloaded the top five ranked AlphaFold models. Per-residue pLDDT scores were extracted from the B-factor field of the PDB files, and mean as well as minimum pLDDT values were summarised separately for the 300–420 surface loop and for the remainder of the protein. The top-ranked model for each lineage was then used for all subsequent structural visualisation and electrostatic analyses. Inspection of rank 2–5 models confirmed that the 300–420 loop is predicted with high local confidence and a similar backbone. Superposition was performed in PyMOL v2.5 (Yuan et al., 2017) via least-squares fitting on conserved backbone residues, and RMSD values were calculated to quantify structural differences. Flexible termini were excluded from alignment to avoid noise. To characterise local structural drift within the loop, residue-wise Cα RMSD values were computed for positions 300–420 between the aligned GPV and MDPV VP1 models.

Intrinsic disorder along the VP1 sequence was predicted using the IUPred3 web server (https://iupred3.elte.hu/) for representative GPV, transition, and MDPV VP1 sequences. Disorder scores were obtained for each residue and used to compare disorder propensity across lineages and to evaluate whether the 300–420 loop exhibits systematically elevated predicted flexibility relative to the remainder of the capsid shell. Normal mode analysis (NMA) was performed on the AlphaFold VP1 models using the bio3d package in R, focusing on the lowest-frequency modes of an elastic network model. For each lineage, mean-square fluctuations were averaged across the lowest modes to obtain residue-wise fluctuation profiles along VP1 and within the 300–420 loop.

Electrostatic surface potentials were computed using pdb2pqr v3.6.0 (AMBER force field, pH = 7.4) and APBS v3.4.1 (Dolinsky et al., 2004), visualized on a color scale of −5 to +5 kT/e (red = negative, blue = positive). The flexible surface loop (residues 300–420) was analyzed using identical orientation and visualization parameters across models. Loop-averaged potentials and residue-level electrostatic differences were then computed to quantify charge redistribution trends. Residue-level electrostatic difference maps for residues 300–420 were obtained by subtracting per-vertex surface potentials between lineages after mapping all VP1 models to a common orientation.

### 2.6 Virus isolation and infection assays

Tissue samples (liver, spleen, kidney, and intestine) were collected from six two-week-old diseased ducks (n = 6) during an acute enteritis outbreak at a commercial duck farm in Jiangsu, China (Ning et al., 2017). Samples were homogenized under sterile conditions, subjected to three freeze–thaw cycles, and clarified by centrifugation. All operations were performed under BSL-2 containment (World Health Organization, 2020).

A 0.2 mL aliquot of the clarified supernatant was inoculated into the allantoic cavity of three 10-day-old SPF duck embryos per sample and serially passaged five times at 37 °C. The harvested allantoic fluid was filtered (0.22 µm) clarified and filtered (0.22 µm) to generate a virus stock, which was titrated in duck embryos and had a titre of 1.8 × 10^6^ ELD_50_/mL. This stock was then used to infect duck embryo fibroblast (DEF) cultures (∼1.2 × 10^6^ cells/well).Once monolayers formed, CPE development was monitored microscopically.

To rule out mixed infections with hemagglutinating viruses (e.g., avian influenza), hemagglutination assays were performed using 1% SPF chicken red blood cells, with AIV H3 and PBS as positive and negative controls, respectively. Viral DNA was extracted, and VP1 was amplified using primers targeting conserved regions. PCR amplicons were verified by gel electrophoresis, purified, sequenced, and identified using NCBI BLAST to confirm lineage (Camacho et al., 2009).

For animal challenge, ten 2-day-old SPF ducklings were randomly assigned to infection and control groups (n = 5 each). The infected group received 0.2 mL of the GPV-type viral suspension (1.8 × 10^6^ ELD_50_/mL) subcutaneously, while controls were injected with PBS. Clinical signs were monitored daily. At the endpoint, tissues were collected for histopathological examination and viral DNA detection. All animal procedures complied with institutional animal care and biosafety regulations and were approved by the local ethics committee (National Academies Council, 2011). The animal challenge experiment was designed as a qualitative pathogenicity assessment. Given the group size (n = 5 per group), outcomes were interpreted descriptively rather than subjected to formal statistical hypothesis testing.

## 3 Results

### 3.1 Phylogenetic inference reveals blurred host boundaries between GPV and MDPV

**FIGURE 1:**
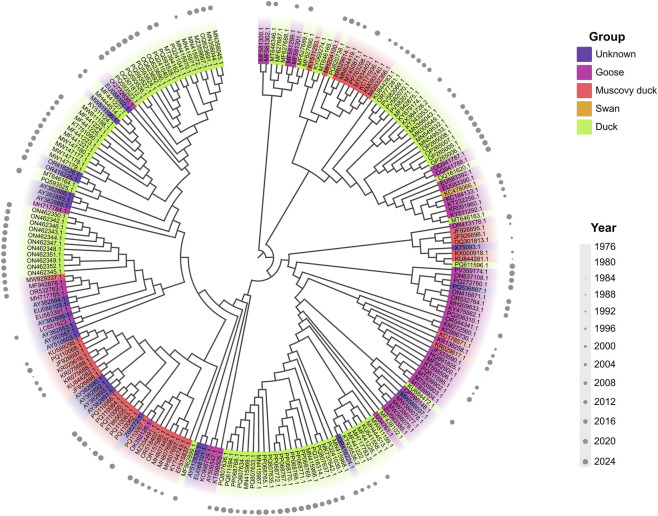
Maximum-likelihood tree of VP1 sequences.

Within the analyzed dataset, the maximum-likelihood phylogeny of VP1 amino acid sequences broadly separated Goose parvovirus (GPV) and Muscovy duck parvovirus (MDPV) lineages, consistent with established taxonomy (Figure 1). However, the division between these host-associated groups was indistinct. Several duck-derived isolates were interspersed within the GPV-associated cluster rather than forming an independent lineage, and a few swan or unclassified isolates were distributed across both major clades.

The phylogenetic topology featured short internal branches and shallow bifurcations, reflecting limited sequence divergence and possible genetic intermixing among host sources. These findings suggest that VP1 evolution in waterfowl parvoviruses proceeds through gradual accumulation of host-related mutations, potentially forming a molecular bridge between goose and duck lineages.

### 3.2 AI-based embedding uncovers a continuous molecular drift across host species

**FIGURE 2:**
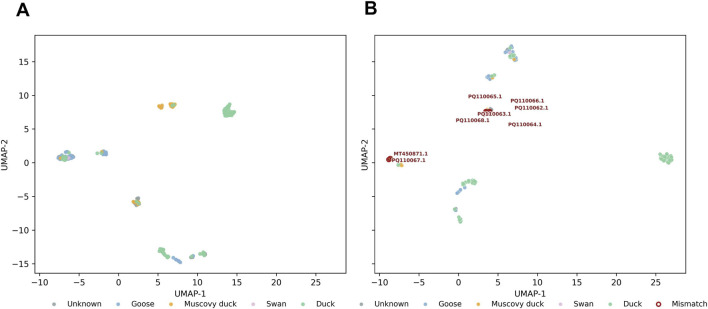
AI-based embedding reveals a continuous host-spectrum topology among VP1 sequences. **(A)** UMAP projection of 1280-dimensional ESM2 embeddings coloured by recorded host origin. Goose, Duck, and Muscovy duck sequences occupy adjacent and partially overlapping regions, forming a continuous molecular trajectory rather than discrete host-restricted clusters. Swan and Unknown sequences localize toward peripheral or intermediate regions of the manifold. **(B)** Isolates with host labels incongruent with their embedding neighborhoods are highlighted (red text and circles), representing candidate interface sequences that may correspond to molecular intermediates of host adaptation or potential annotation errors. Axes correspond to the two-dimensional UMAP projection (UMAP-1 and UMAP-2) of the ESM2 embedding space. The spatial orientation of **(A,B)** varies due to the stochastic optimization process inherent to UMAP; nevertheless, the underlying manifold topology and inter-cluster relationships remain consistent across projections.

To probe molecular relationships that exceed the resolution of traditional phylogenetic analysis, we applied an AI-based protein language model (ESM2) to generate VP1 sequence embeddings. Figure 2A shows the global UMAP projection of all VP1 embeddings, colored by recorded host category to visualize the overall host-spectrum topology, whereas Figure 2B displays the same embedding with interface isolates highlighted to emphasize sequences that lie between the goose- and duck-associated regions or whose recorded host labels are incongruent with their local embedding neighborhoods.

In contrast to the bifurcated topology of the phylogenetic tree, the ESM2 embedding displayed a smooth, continuous distribution of VP1 sequences across host species (Figure 2A). When visualized in UMAP space, sequences from goose, duck, and Muscovy duck occupied partially overlapping regions rather than forming distinct clusters, indicating that the embedding captures gradual molecular similarity among host-associated isolates. Swan and unclassified (“Unknown”) sequences appeared near the periphery of the embedding manifold, consistent with limited sampling or intermediate molecular affinity to the main host groups.

A subset of isolates lay at the interface between the goose- and duck-associated regions (Figure 2B). Among these, MT450871.1 and PQ110062–PQ110068 occupied positions inconsistent with their recorded host labels, suggesting cross-host molecular resemblance or potential annotation errors. These interface isolates delineate a transition zone between host-associated regions, highlighting gradual molecular shifts that remain unresolved in classical tree-based analyses. These isolates were therefore selected for subsequent structural and functional investigation.

Taken together, the embedding results indicate that VP1 diversity across goose, duck, and Muscovy duck isolates forms a continuous molecular trajectory rather than discrete host-restricted lineages. Waterfowl parvoviruses thus exhibit graded molecular transitions across hosts, implying that host specificity evolves along a continuum rather than through abrupt host shifts.

To confirm that the embedding-derived continuum represents a genuine molecular signal and not a visualization artifact, we next conducted quantitative analyses of its topology and host-association stability.

### 3.3 Quantitative embedding analysis confirms continuous host-spectrum topology

**FIGURE 3:**
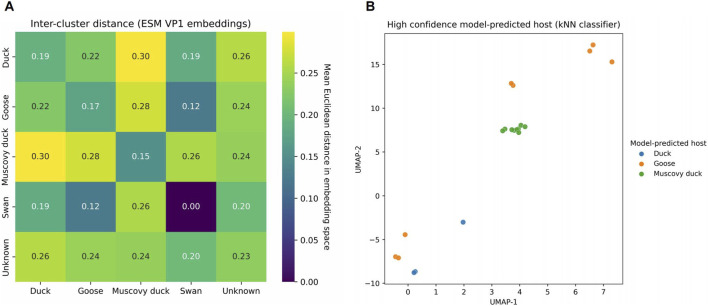
Quantitative validation of host-spectrum topology and embedding-based classification stability. **(A)** Heatmap of mean pairwise Euclidean distances between host groups in the VP1 embedding space derived from ESM2. The colour scale reports the mean Euclidean distance in embedding space, expressed in arbitrary units. **(B)** UMAP projection of VP1 embeddings for sequences with high-confidence predictions, coloured by model-predicted host (kNN classifier). Axes correspond to the two-dimensional UMAP projection of 1280-dimensional ESM2 embeddings. Differences in global orientation between panels arise from the stochastic optimization process of UMAP but do not affect the underlying manifold topology or relative inter-cluster relationships.

To quantify relationships within the embedding space, we computed pairwise inter-group distances among host-labeled sequences using L2-normalized vectors from the original ESM2 representation (Figure 3A). Across all host pairs, normalized inter-group distances ranged from 0.12 to 0.30. Among the three main domestic waterfowl hosts (goose, duck, Muscovy duck), distances between goose and duck isolates were the smallest (mean normalized Euclidean distance = 0.22), indicating close molecular proximity. Thus, the larger distances involving Muscovy duck isolates (0.26–0.30) represent roughly a 20%–35% increase relative to the goose–duck pair (0.22), consistent with their more peripheral position in the embedding map and supporting that goose and duck isolates occupy the closest molecular neighborhood among the main host groups. In contrast, Muscovy duck sequences were more distinct from both goose and duck (0.28 for goose–Muscovy duck and 0.30 for duck–Muscovy duck), while swan and unclassified (“Unknown”) isolates occupied intermediate or peripheral positions, likely reflecting limited sampling and mixed host metadata. These normalized Euclidean distances are intended as comparative descriptors of global molecular proximity in the embedding space rather than as formal statistical tests; the modest difference between 0.22 and 0.28–0.30 nevertheless indicates that goose and duck isolates occupy the closest molecular neighborhood among domestic waterfowl hosts. These spatial patterns support a graded molecular topology rather than sharply divided clusters among host groups.

To evaluate structural stability, a *k*-nearest-neighbor classifier (*k* = 5) was trained on the high-dimensional embeddings. Predictions with high confidence (probability ≥0.85) formed compact, well-defined regions on the UMAP map (Figure 3B), demonstrating that host-associated molecular features are consistently encoded within the manifold. The close spatial proximity between goose and duck clusters mirrored the small inter-group distances observed in Figure 3A, underscoring that host differentiation follows a smooth gradient rather than discrete divisions.

The convergence of quantitative distance metrics and classification stability provides strong evidence that the ESM2 embedding preserves a coherent host-associated continuum. Within this framework, the goose–duck relationship emerges as the closest molecular neighborhood among waterfowl parvoviruses, defining a quantitative bridge across the host-adaptation landscape.

### 3.4 Structural and dynamical remodeling of the VP1 surface loop along the GPV–MDPV continuum

**FIGURE 4:**
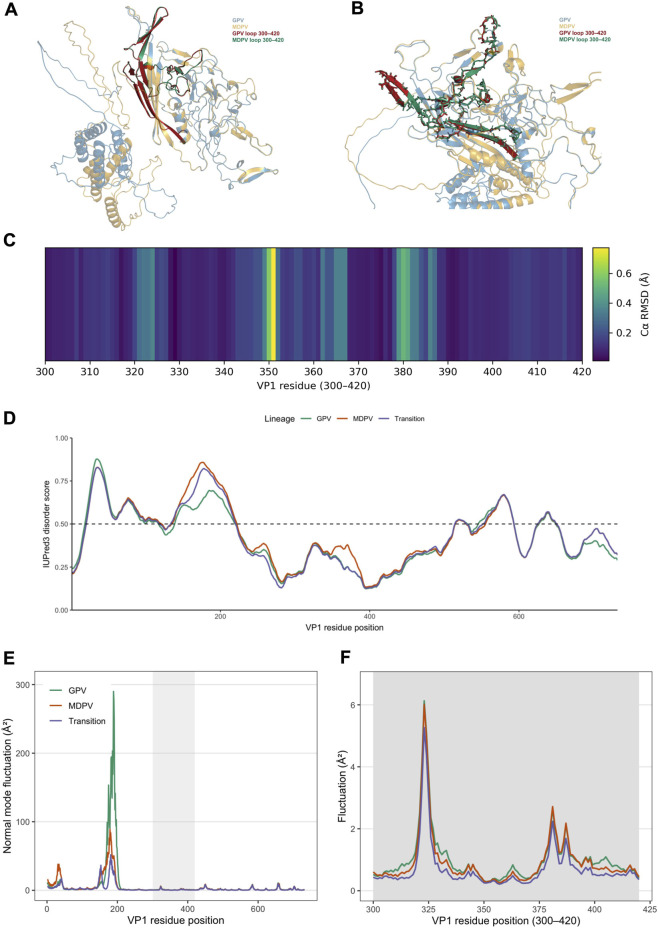
Structural and dynamical remodeling of the VP1 capsid loop along the GPV–MDPV continuum. **(A)** Superposition of VP1 structures from GPV (blue) and MDPV (orange). The surface-exposed VP1 loop (residues 300–420) is highlighted in red for GPV and green for MDPV, as indicated in the in-panel colour legend, whereas the remainder of the capsid is shown in pale blue/orange. **(B)** Close-up view of the VP1 loop region (residues 300–420) after structural superposition. The 300–420 loop is shown as a thicker ribbon in red (GPV) and green (MDPV), while the surrounding capsid backbone is rendered in pale blue/orange. This region concentrates most lineage-specific substitutions and exhibits the largest conformational differences between GPV and MDPV. **(C)** Heatmap of residue-wise Cα RMSD (Å) between the aligned GPV and MDPV VP1 models, restricted to residues 300–420. Most positions within the loop show low RMSD values, whereas two localized segments around residues ∼350 and ∼380 exhibit elevated deviations, indicating that structural differences are focused on specific subregions rather than reflecting a wholesale rearrangement of the capsid fold. **(D)** Intrinsic disorder profiles of VP1 predicted with IUPred3 for representative GPV (teal), MDPV (orange) and transition (purple) sequences. The y-axis shows the disorder score (0–1); the dashed horizontal line marks the classical 0.5 threshold. VP1 is overall well ordered, but the 300–420 loop displays consistently higher disorder propensity than the surrounding capsid shell, with slightly increased scores for the transition and MDPV sequences relative to GPV. **(E)** Normal-mode fluctuations of VP1 obtained from elastic-network normal mode analysis (bio3d) of the AlphaFold models. Plotted are residue-wise mean-square fluctuations (Å^2^) averaged over the lowest-frequency modes for GPV (teal), MDPV (orange) and the transition isolate (purple). Several mobile surface regions are apparent; the 300–420 loop (grey shading) emerges as one of the comparatively flexible segments on the capsid surface. **(F)** Zoom-in of the normal-mode fluctuation profiles for the 300–420 loop region. In this loop, GPV shows the lowest predicted mobility, the transition isolate exhibits intermediate fluctuations, and MDPV shows the highest amplitudes, particularly in the same subregions that display elevated Cα RMSD in **(C)**. This pattern is consistent with a gradual increase in local flexibility along the GPV–transition–MDPV continuum.

We next examined whether the embedding-derived molecular continuum corresponds to structural and physicochemical remodeling in the VP1 capsid protein. Representative VP1 structures from classical GPV and MDPV—selected as the two extremes of the continuum—were modeled using AlphaFold for comparative analysis (Figure 4A). The overall capsid architecture remained highly conserved, which underscores the strong structural constraints characteristic of the Parvoviridae family and suggests that adaptive changes are likely confined to localized, surface-exposed regions. Against this conserved background, a distinct deviation emerged in the flexible surface loop spanning residues 300–420 (Figure 4B). In MDPV, this loop adopted an open, outward-oriented conformation, whereas in GPV it appeared more compact and recessed.

Consistent with our AlphaFold confidence analysis (see Methods), the 300–420 surface loop is predicted with high local confidence across rank 1–5 models for GPV, the transition isolate, and MDPV (mean pLDDT ≈96.5–96.7, minimum per-residue pLDDT ≥75.9), supporting the reliability of the inferred loop conformations in this region. To quantify the local structural differences between GPV and MDPV, we computed residue-wise Cα RMSD values for residues 300–420 between the aligned VP1 models (Figure 4C). This analysis revealed that most positions exhibit low RMSD, whereas two subregions centered around ∼350 and ∼380 display elevated RMSD, indicating that the structural drift is concentrated in specific segments rather than reflecting a wholesale rearrangement of the capsid fold.

To assess whether this loop also differs in predicted conformational flexibility, we analyzed intrinsic disorder along VP1 using IUPred3 for GPV, the transition isolate, and MDPV (Figure 4D). Disorder scores remained mostly below the classical 0.5 threshold across the protein, indicating that VP1 is globally well ordered, but the 300–420 loop consistently showed modestly elevated scores relative to the surrounding shell, consistent with a flexible surface-exposed element. Notably, the transition and MDPV sequences exhibited slightly higher disorder propensity than GPV at the center of the loop, suggesting that host-associated sequence drift is accompanied by a subtle increase in local conformational plasticity.

We next probed large-scale motions of VP1 using normal mode analysis (NMA) based on the AlphaFold models (Figures 4E,F). Overall mean-square fluctuations along the lowest-frequency modes highlighted several mobile surface regions, with the 300–420 loop emerging as a comparatively flexible segment on the capsid surface (Figure 4E). When we zoomed in on residues 300–420 (Figure 4F), GPV displayed the lowest fluctuation amplitude, the transition isolate showed an intermediate profile, and MDPV exhibited the highest predicted mobility, particularly in the segments that also showed elevated Cα RMSD. Taken together, these observations indicate that the GPV–transition–MDPV trajectory is accompanied by coordinated changes in loop conformation, local flexibility, and intrinsic disorder, consistent with a structurally and dynamically tunable module mediating host adaptation.

### 3.5 Electrostatic fine-tuning of the VP1 loop aligns with the embedding-derived host continuum

**FIGURE 5:**
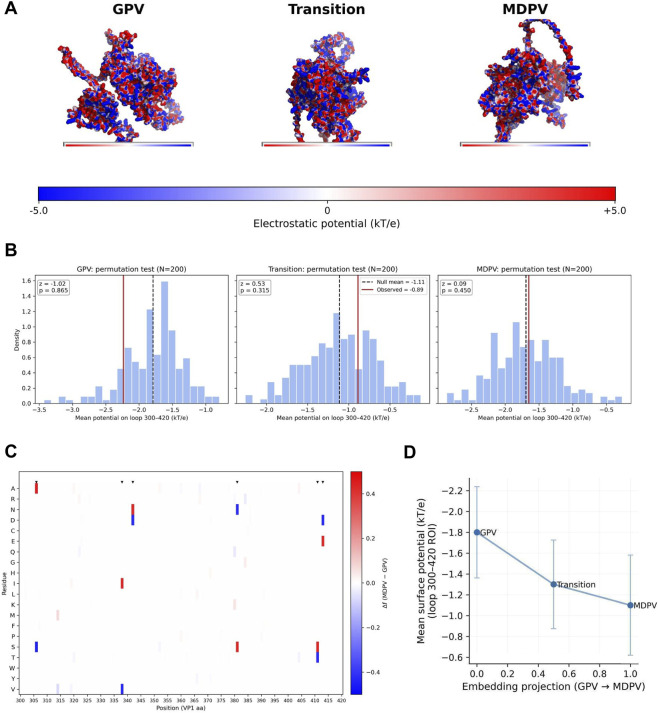
Electrostatic fine-tuning of the VP1 300–420 loop along the embedding-derived host continuum. **(A)** Electrostatic surface potential maps (−5 to +5 kT/e) for representative GPV, transition and MDPV VP1 models computed with pdb2pqr/APBS. The colour scale reports surface potential in units of kT/e. The maps reveal a gradual redistribution of charge across VP1, with the 300–420 loop becoming progressively more neutral along the GPV → transition → MDPV trajectory. **(B)** Permutation tests (n = 200) of loop-averaged surface potentials for the 300–420 region. Histograms show the permutation-derived null distributions, and vertical lines indicate the observed mean potentials. All three lineages fall within their empirical uncertainty intervals, but their ordering is consistently monotonic (GPV < transition < MDPV), indicating a stable trend toward surface neutralisation. **(C)** Residue-level amino acid frequency differences (Δf = frequency(MDPV) − frequency(GPV)) across the VP1 300–420 loop, highlighting charge-changing substitutions concentrated in this region. Most large differences correspond to distributed Lys→Asp/Glu replacements at multiple positions, consistent with a diffuse, cumulative change rather than a single focal substitution. **(D)** Relationship between the embedding-derived continuum coordinate and the mean surface potential of the VP1 300–420 loop. Points represent GPV, transition and MDPV; error bars indicate the standard deviation of the permutation-derived null distributions. The near-linear association shows that AI-derived embedding drift corresponds quantitatively to measurable electrostatic remodeling of VP1, while remaining descriptive rather than implying a specific causal mechanism.

Having established that the embedding-derived continuum is anchored in a specific, structurally coherent VP1 loop, we next asked whether these conformational differences are coupled to electrostatic remodeling. Surface potentials were computed for representative GPV, transition, and MDPV models using pdb2pqr and APBS (Figure 5A). The resulting maps revealed a progressive redistribution of charge across the 300–420 loop: GPV displayed predominantly positive electrostatic patches, the transition isolate exhibited mixed potentials, and MDPV presented a nearly neutral or slightly negative surface. Such a steady shift toward neutrality parallels the molecular continuum inferred from embeddings, and suggests that electrostatic fine-tuning of this loop may contribute to host-associated divergence, although these analyses do not provide direct evidence on receptor binding or usage.

For quantitative comparison, loop-averaged surface potentials were compared among the three models (Table 1). Although mean values overlapped with permutation-derived null distributions (n = 200; Figure 5B), their relative ordering—GPV (−1.81 kT/e), Transition (−1.34 kT/e), and MDPV (−1.16 kT/e)—displayed a consistent monotonic trend toward electrostatic neutralization. Rather than treating these comparisons as formal hypothesis tests, we interpret them as effect sizes relative to the empirical null, which emphasises direction and magnitude over strict statistical significance. While absolute differences were subtle, their directional coherence was striking, reinforcing the notion of a stable, cumulative drift rather than stochastic variation. Pairwise comparisons yielded small yet reproducible effect sizes (small effect sizes Cohen’s d < 0.1), with detailed statistics provided in Table 2.

**TABLE 1:** Loop-averaged surface electrostatic potentials (residues 300–420) of representative VP1 structures.

Group	Mean potential (kT/e)	95% CI
GPV	−1.807	[–2.790, −0.913]
Transition	−1.337	[–1.943, −0.825]
MDPV	−1.164	[–2.172, −0.109]

Electrostatic potentials were computed using APBS (v3.4.1) at pH 7.0 and 150 mM ionic strength. Values represent the mean potential across the surface-exposed loop region for each structure.

**TABLE 2:** Pairwise comparisons of electrostatic potentials.

Pairwise comparison (A–B)	Δ mean (A–B, kT/e)	95% CI	Cohen’s d	95% CI
Transition – MDPV	−0.172	[–1.414, 0.975]	−0.017	[–0.123, 0.108]
Transition – GPV	+0.470	[–0.615, 1.591]	+0.051	[–0.069, 0.164]
MDPV – GPV	+0.642	[–0.713, 2.046]	+0.054	[–0.066, 0.159]

Values represent Mean ± SD, of electrostatic potential differences (kT/e) computed from 200 permutation replicates. Positive values indicate relative surface neutralization compared with the reference GPV, model.

Residue-level differential potential maps localized the main charge transitions to the 300–420 loop (Figure 5C). The shifts were primarily driven by distributed Lys→Asp/Glu substitutions across multiple positions, which together produce a diffuse, cumulative electrostatic change rather than a single focal event. In doing so, these substitutions modulate the electrostatic properties of a surface-exposed loop that, by analogy to other parvoviruses, is likely involved in host interactions, raising the possibility that they influence receptor engagement or host range. At present, however, this remains a mechanistic hypothesis, as we do not yet have direct receptor-binding, docking, or infection assays with defined receptors.

When loop-averaged potentials were projected onto the embedding axis, an approximately linear correlation emerged between the embedding coordinate and surface potential (Figure 5D). This near-linearity indicates that the embedding-derived continuum reflects systematic variation in VP1 surface electrostatics and provides a quantitative link between the abstract geometry of AI-derived embeddings and tangible biophysical properties; nonetheless, this relationship should be interpreted as correlative rather than as direct evidence for a specific causal mechanism, such as altered receptor affinity.

Together, these analyses—spanning sequence embeddings, three-dimensional conformation, and electrostatic potential profiles are consistent with a coherent biophysical continuum associated with host adaptation. The progressive shift toward surface neutralization (GPV → Transition → MDPV) suggests a plausible mechanistic link between molecular representation and host-range evolution in waterfowl parvoviruses. In practice, the loop’s gradual neutralization defines a compact and computationally traceable candidate marker of cross-host potential, suggesting that isolates trending toward the MDPV-like electrostatic signature may represent early molecular correlates of host adaptation, a testable prediction that will require future functional and receptor-based experiments to evaluate.

### 3.6 Infection validation in embryos, cells, and animals

To determine whether the molecular–electrostatic continuum manifests in biological function, infection assays were performed in embryos, cultured cells, and live animals. A GPV-type strain was isolated and molecularly identified from diseased ducks during a natural outbreak. The isolate shared >99.8% nucleotide identity with classical GPV reference strains (Table 3), and hemagglutination testing excluded contamination by other hemagglutinating viruses.

**TABLE 3:** Sequence identity of the isolated GPV-type strain.

Description	Scientific name	Max score	Query cover	E-value	Percent identity	Accession length	Accession
Goose parvovirus strain DY16, complete genome	Goose parvovirus	3,977	100%	0.0	100%	5,046	MH209633.1
Goose parvovirus strain RC16, complete genome	Goose parvovirus	3,977	100%	0.0	100%	5,046	KY475562.1
Goose parvovirus strain DX, complete genome	Goose parvovirus	3,911	100%	0.0	99.95%	5,046	OR532764.1
Goose parvovirus isolate BD, complete genome	Goose parvovirus	3,911	100%	0.0	99.95%	5,046	ON416871.1
Goose parvovirus strain XT, complete genome	Goose parvovirus	3,980	100%	0.0	99.86%	5,102	OR532763.1
Goose parvovirus isolate HB-DX, complete genome	Goose parvovirus	3,980	100%	0.0	99.86%	5,046	OR544341.1
Goose parvovirus strain RC16, complete genome	Goose parvovirus	3,975	100%	0.0	99.82%	5,106	ON637108.1

BLAST, analysis of the VP1 gene confirmed that the duck-origin isolate shares >98% nucleotide identity with classical GPV, strains, verifying its assignment to the GPV, lineage (GenBank, accessed May 2025).

**FIGURE 6:**
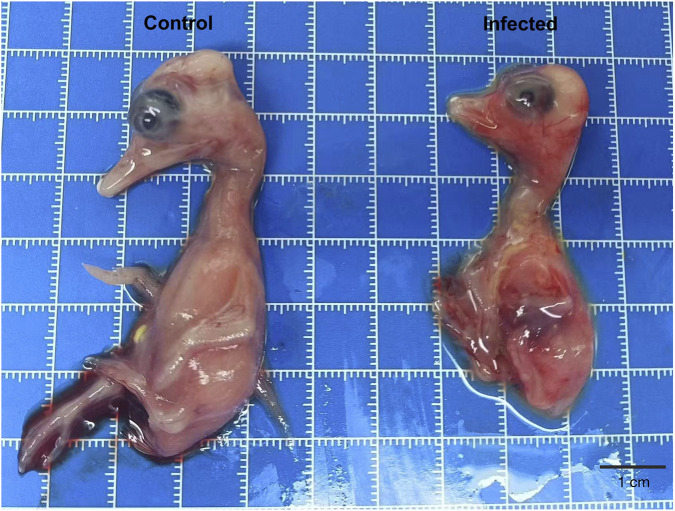
Duck embryos infected with the GPV-type isolate showed vascular congestion and growth retardation relative to controls.

Inoculation of 10-day-old SPF duck embryos with 0.2 mL of a GPV-type virus suspension (1.8 × 10^6^ ELD_50_/mL) led to vascular congestion and growth retardation compared with controls (Figure 6). No hemagglutination activity was detected in the allantoic fluid, consistent with the non-hemagglutinating nature of GPV. These observations show that, at this defined infectious dose, the duck-derived isolate can productively infect and cause disease in duck embryos.

**FIGURE 7:**
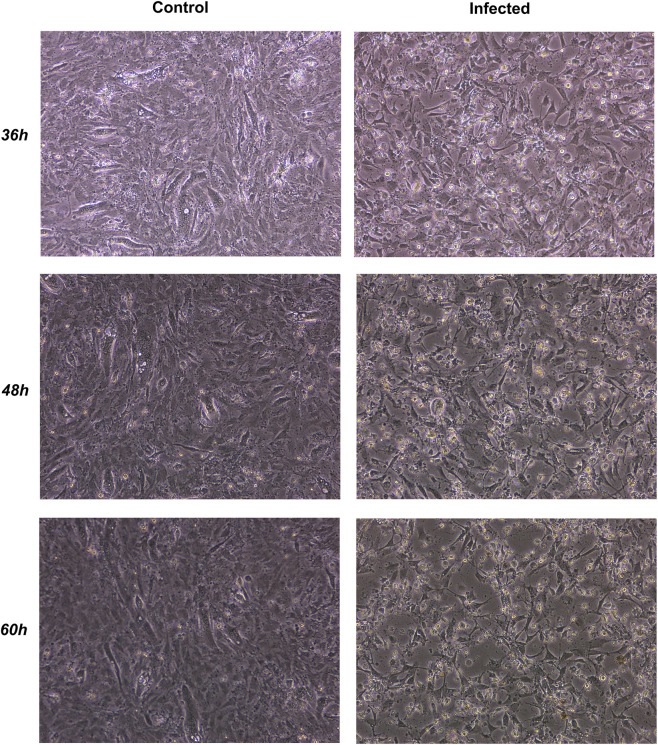
DEF cells displayed progressive cytopathic effects (CPE) at 36–60 hpi, including rounding and detachment.

At the cellular level, duck embryo fibroblast (DEF) cultures infected with fifth-passage virus were monitored at 36, 48, and 60 h post-infection (hpi). Compared with uninfected controls, infected cells showed progressive cytopathic effects (CPE)—cell rounding, shrinkage, detachment, and disruption of the monolayer (Figure 7). Morphological changes appeared at 36 hpi, intensified by 48 hpi, and culminated in extensive granulation and detachment at 60 hpi, consistent with active replication and lytic activity in duck-derived cells. These assays therefore provide qualitative confirmation that, when applied at a titrated infectious dose, the isolate can infect DEF monolayers and reproducibly induce progressive CPE.

For *in vivo* validation, ducklings were experimentally infected after confirming replication in embryos and DEF cells. During the first 24 h post-inoculation, no clinical symptoms were noted. Between 24 and 36 hpi, infected ducklings developed mild diarrhea and reduced appetite. By 60 hpi, diarrhea became severe, accompanied by lethargy, ruffled feathers, and decreased activity. Around 72 hpi, partial mortality occurred, and several birds exhibited intermittent tremors and neck twisting, suggesting possible central nervous system involvement. Control ducklings remained normal and active throughout.

**FIGURE 8:**
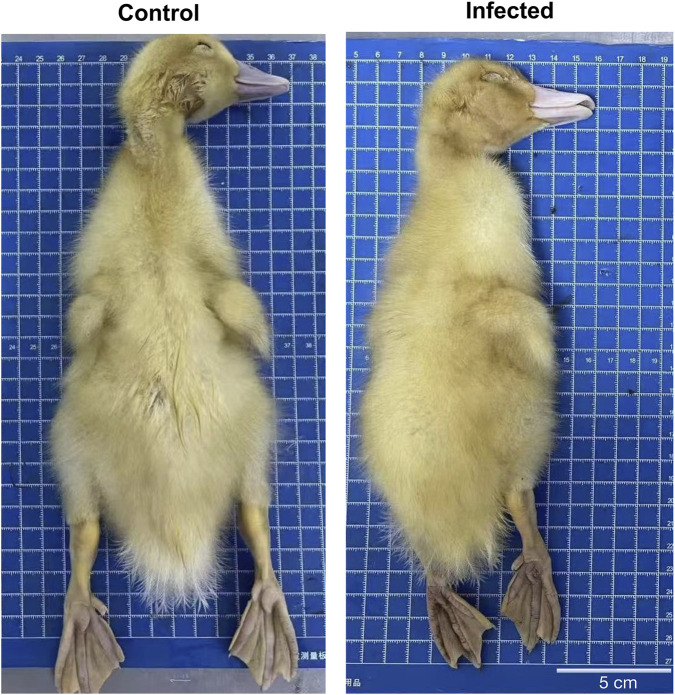
Gross appearance of control and infected ducklings. Whole ducklings from the control (left) and infected (right) groups photographed on a 1 cm × 1 cm grid board. Infected ducklings appear smaller, paler, and less well-feathered than controls. On-panel labels indicate the experimental groups (“Control,” “Infected”). Scale bar: 5 cm.

**FIGURE 9:**
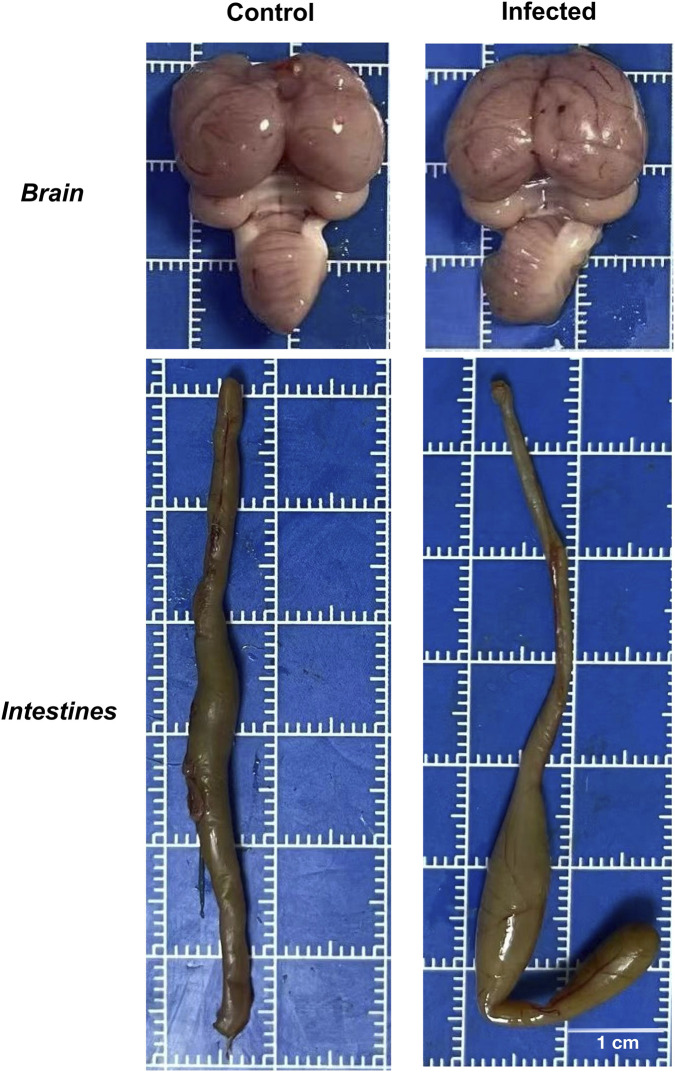
Gross pathological lesions in brains and intestines of infected ducklings. Brains (top row) and intestines (bottom row) from control (left) and infected (right) ducklings photographed on a 1 cm × 1 cm grid board. Infected brains show distinct branch-like haemorrhages and mild surface congestion compared with smooth control brains. Infected intestines exhibit wall thinning, mucosal sloughing and focal collapse, whereas control intestines maintain a smooth, intact mucosal surface. On-panel labels indicate the experimental groups. Scale bar: 1 cm. These gross pathological changes are consistent with partial host adaptation and active viral replication observed in embryonic and cellular assays.

Gross pathology revealed that infected ducklings were smaller and paler than controls (Figure 8). Brains were slightly reduced in size and showed branching hemorrhages and mild congestion (Figure 9). The most prominent lesions were observed in the intestines, characterized by wall thinning, mucosal sloughing, and focal collapse, whereas control intestines appeared smooth and intact (Figure 9).

Overall, these embryonic, cellular, and animal results collectively show that the duck-derived GPV isolate can infect, replicate, and induce characteristic lesions in ducks, providing qualitative biological support for partial host adaptation along the molecular continuum.

## 4 Discussion

Viral adaptation is not governed by a single universal mode but can follow distinct evolutionary patterns depending on the virus and its ecological context. In rapidly evolving RNA viruses such as SARS-CoV-2, several studies have shown that structural proteins can accumulate mutations piecemeal over time, with these changes later coalescing into larger constellations in variants of concern (Ali and Caetano-Anollés, 2024a; Ali and Caetano-Anollés, 2024b). In the specific case of the small, non-enveloped DNA viruses studied here, our analyses suggest that host adaptation in waterfowl parvoviruses can proceed along a smoother, continuous trajectory—supported not only by statistical inference but also by structural and biological validation. By integrating AI-derived molecular embeddings, structural biophysics, and infection assays, we find that the Goose parvovirus (GPV) and Muscovy duck parvovirus (MDPV) lineages occupy a continuous molecular spectrum of host adaptation within this system. Within this constrained biophysical and evolutionary context, this perspective reframes viral evolution as a gradual optimization process occurring within a continuous biophysical landscape rather than as a sequence of sharp host shifts (Tomaszewski et al., 2023).

Traditional phylogenetic trees describe evolutionary space as a branching hierarchy. While effective for identifying major lineage divergences, this framework often masks subtle adaptive transitions, particularly when sequence divergence is minimal (Rothenburg and Brennan, 2020). Protein language model (PLM) embeddings, by contrast, preserve molecular continuity in high-dimensional feature space, allowing gradual transitions to emerge as smooth trajectories. Within this manifold, GPV and MDPV sequences occupy adjacent and partially overlapping domains, together forming a molecular gradient that connects sequence variation with host range (Yang and Rao, 2021). The persistence of this topology across different random initializations and dimensionality-reduction parameters supports the view that it reflects an underlying molecular signal rather than a visualization artifact.

Structural and electrostatic analyses provide a mechanistic basis for this continuum. Comparative modeling revealed that a flexible surface-exposed loop (residues 300–420) in the VP1 capsid protein undergoes progressive remodeling along the GPV–MDPV axis. This loop displays marked conformational flexibility and a gradual reduction in positive surface charge, driven by distributed Lys → Asp/Glu substitutions at multiple positions. These adjustments are consistent with a fine-tuning mechanism in which small structural and electrostatic shifts modulate the properties of receptor- or immune-exposed surfaces, although direct evidence for altered receptor affinity is currently lacking (Hall et al., 2013). From an applied standpoint, the loop’s gradual neutralization defines a compact and computationally traceable marker of cross-host potential, suggesting that isolates trending toward MDPV-like electrostatic signatures may represent early molecular correlates of increased cross-host compatibility. The approximately monotonic relationship between embedding coordinates and loop electrostatic potential quantitatively connects the latent space of AI-derived molecular embeddings to explicit biophysical observables, providing a link between embeddings and structural observables in this system (Amorim, 2019).

Beyond these electrostatic trends, our structural and dynamical analyses converge on the same VP1 surface loop (residues ∼300–420) as a tunable module along the GPV–MDPV continuum. AlphaFold models indicate that the global capsid fold is highly conserved, whereas residue-wise Cα RMSD profiles show that structural divergence is locally concentrated within this loop rather than distributed across the capsid. IUPred3 disorder scores and normal mode analysis further reveal that the 300–420 region is both more flexible and more dynamically mobile than the surrounding shell, with GPV exhibiting the lowest predicted mobility, the transition isolate an intermediate profile, and MDPV the highest fluctuations. Taken together, these observations support a model in which host-associated evolution in this system acts not by globally rewiring the capsid, but by subtly tuning the conformation, intrinsic disorder, and large-scale motions of a single exposed loop that appears to play a central role in shaping cross-host compatibility in this system.

The location and biophysical profile of this loop—surface exposed, relatively flexible, and undergoing progressive electrostatic neutralization—also make it an attractive candidate for modulating receptor engagement or co-factor binding. However, our current data do not allow us to assign a specific binding role based on *in silico* evidence alone. We therefore deliberately refrain from interpreting the loop as a defined receptor-binding site at this stage and instead highlight it as a high-priority target for future work that combines dedicated binding-site prediction tools with experimental receptor-binding and neutralization assays.

Experimental infection assays further close the loop between molecular inference and biological function. A GPV-type isolate obtained from diseased ducks replicated in duck embryos and duck embryo fibroblast (DEF) cells and produced characteristic pathological lesions in ducklings. The overall pattern of disease and tissue involvement resembled that of classical GPV infections in geese, suggesting functional similarity despite host differences. These findings demonstrate that a virus genetically aligned with classical GPV can infect and replicate in ducks, providing qualitative biological support for the predicted molecular continuum (Agudelo-Romero et al., 2008).

Conceptually, this continuum highlights the dual character of viral evolution: rigid conservation of structural frameworks coupled with flexible electrostatic adaptation. GPV-lineage genomes maintain their overall capsid architecture while navigating local energetic landscapes that permit incremental adjustments in host compatibility. Such fine-tuning of surface charge and conformation is therefore best viewed as a candidate mechanism for host-range modulation in compact, non-enveloped DNA viruses with highly constrained capsid architectures—such as parvoviruses and, potentially, related adeno-associated and circoviruses—rather than as a general rule across all viral families (Wei et al., 2019; Tan et al., 2024).

Beyond waterfowl parvoviruses, the molecular–electrostatic continuum proposed here is conceptually most relevant to other small, non-enveloped DNA viruses with compact capsids and surface-loop–mediated receptor interactions. Members of Parvoviridae, Circoviridae and adeno-associated viruses share a similar architectural logic: a highly conserved capsid core decorated by exposed loops that concentrate sequence variation, mediate receptor binding and immune evasion, and can alter host range or tissue tropism through relatively modest local changes rather than wholesale remodeling of the capsid scaffold. Structural and functional work in these systems has repeatedly implicated gradual remodeling of surface loops as a plausible route to modulating receptor usage or host specificity (Wei et al., 2019). In this sense, continuum-like trajectories of host adaptation may recur within structurally constrained DNA virus families, even though they are not expected to generalize to RNA viruses or large DNA viruses that evolve through more episodic, recombination-driven dynamics. Our study therefore provides a concrete case in which such a continuum can be quantified and mechanistically dissected in a representative compact DNA virus lineage, while also highlighting a broader class of viruses for which similar embedding-guided analyses may be informative.

Even with this broader conceptual relevance, the scope of our framework remains inherently limited. Our data directly concern only small, non-enveloped DNA viruses with compact capsids. Other viral groups, particularly RNA viruses and large DNA viruses that evolve through rapid mutation, recombination, and complex immune modulation, may follow more episodic and discontinuous evolutionary dynamics. In RNA viruses such as SARS-CoV-2, for example, previous work has shown that mutations in structural proteins can accumulate piecemeal over time and are progressively recruited into larger constellations in variants of concern (Ali and Caetano-Anollés, 2024a; Ali and Caetano-Anollés, 2024b). This behaviour, while also reflecting a form of continuity in mutational and structural space, differs from the comparatively smooth, low-dimensional VP1 continuum described here at the level of a single capsid loop. For such systems, embedding-based molecular manifolds may still be informative, but they are not expected *a priori* to represent host transitions as low-dimensional, quasi-linear trajectories and would require dedicated, system-specific evaluation. Accordingly, we regard the continuum model proposed here as a working hypothesis that is presently best justified for compact DNA viruses with conserved capsid cores, and we view any extension to other viral families as speculative until supported by direct empirical evidence.

This study represents an initial step toward quantifying viral adaptation as a continuous, measurable process. Our embedding analysis was limited to the VP1 capsid protein, whereas non-structural genes such as NS1 and Rep likely contribute to host specificity through replication and immune-evasion functions. Moreover, current PLM embeddings (e.g., ESM2) were trained on general protein corpora rather than on parvovirus-specific data, potentially limiting resolution at the host level. The structural and electrostatic analyses are based on static models; future integration of cryo-electron microscopy, receptor-binding assays, and molecular dynamics simulations will be important to more fully capture conformational dynamics. Finally, our biological validation was limited to a single GPV-type isolate, and broader temporal and geographic sampling will be essential to assess whether this molecular continuum represents a recurrent adaptive trajectory for waterfowl parvoviruses.

Viewed jointly, our results support the view that AI-derived molecular embeddings effectively bridge the gap between sequence statistics and mechanistic biology in this system. By tracing a continuous path from sequence space to structure, and from structure to infectivity, we provide a quantitative and interpretable framework for understanding viral host adaptation. This embedding-guided approach may help shift the focus from purely descriptive genomics toward more predictive modeling, and in our data it is consistent with the idea that continuous molecular drift—rather than abrupt host jumps—may play a major role in host-spectrum expansion in the compact DNA viruses analysed here. Thus, what appears as a phylogenetic dichotomy may, at least in this parvovirus system, be better described as a continuum of adaptive drift, rendering host shifts less abrupt and more predictable than they seem from tree-based representations alone. Future integration of embedding-guided mutational scanning, receptor-binding measurements, and longitudinal surveillance could yield predictive indicators of cross-host potential, linking data-driven molecular semantics with the physical principles governing viral emergence. In parallel, virus-specific or fine-tuned protein language models could further enhance host-resolution accuracy, enabling more precise prediction of cross-species emergence from sequence data.

## Data Availability

All data and code used in this study are available at https://github.com/ShaoNihui/gpv-hostadaptation-embeddings.git. The repository contains the input datasets (alignments, embeddings, distance matrices, structural input files), all custom analysis scripts, and the Conda environment file (environment.yml) specifying the software versions used, and is provided for community use and reference.
