# Correction: Protein embeddings reveal a continuous molecular landscape of host adaptation in waterfowl parvoviruses

**DOI:** 10.3389/fbinf.2026.1839097

**Published:** 2026-04-16

**Authors:** Nihui Shao, Yunfei Guo

**Affiliations:** 1 Faculty of Science, University of Bern, Bern, Switzerland; 2 Animal Infectious Disease Laboratory, School of Veterinary Medicine, Yangzhou University, Yangzhou, China

**Keywords:** host adaptation, molecular continuum, protein language model, structural–electrostatic remodeling, VP1 protein, waterfowl parvovirus

The Data Availability statement was erroneously given as “The raw data supporting the conclusions of this article will be made available by the authors, without undue reservation.”

The correct Data Availability statement is “All data and code used in this study are available at https://github.com/ShaoNihui/gpv-hostadaptation-embeddings.git. The repository contains the input datasets (alignments, embeddings, distance matrices, structural input files), all custom analysis scripts, and the Conda environment file (environment.yml) specifying the software versions used, and is provided for community use and reference.”

The original version has been updated.

