# Supplementary figures and images for: Protein embeddings reveal a continuous molecular landscape of host adaptation in waterfowl parvoviruses
Source: Front Bioinform. 2026 Jan 27;5:1738737. doi: 10.3389/fbinf.2025.1738737 (PMC12887590; doi:10.3389/fbinf.2025.1738737)

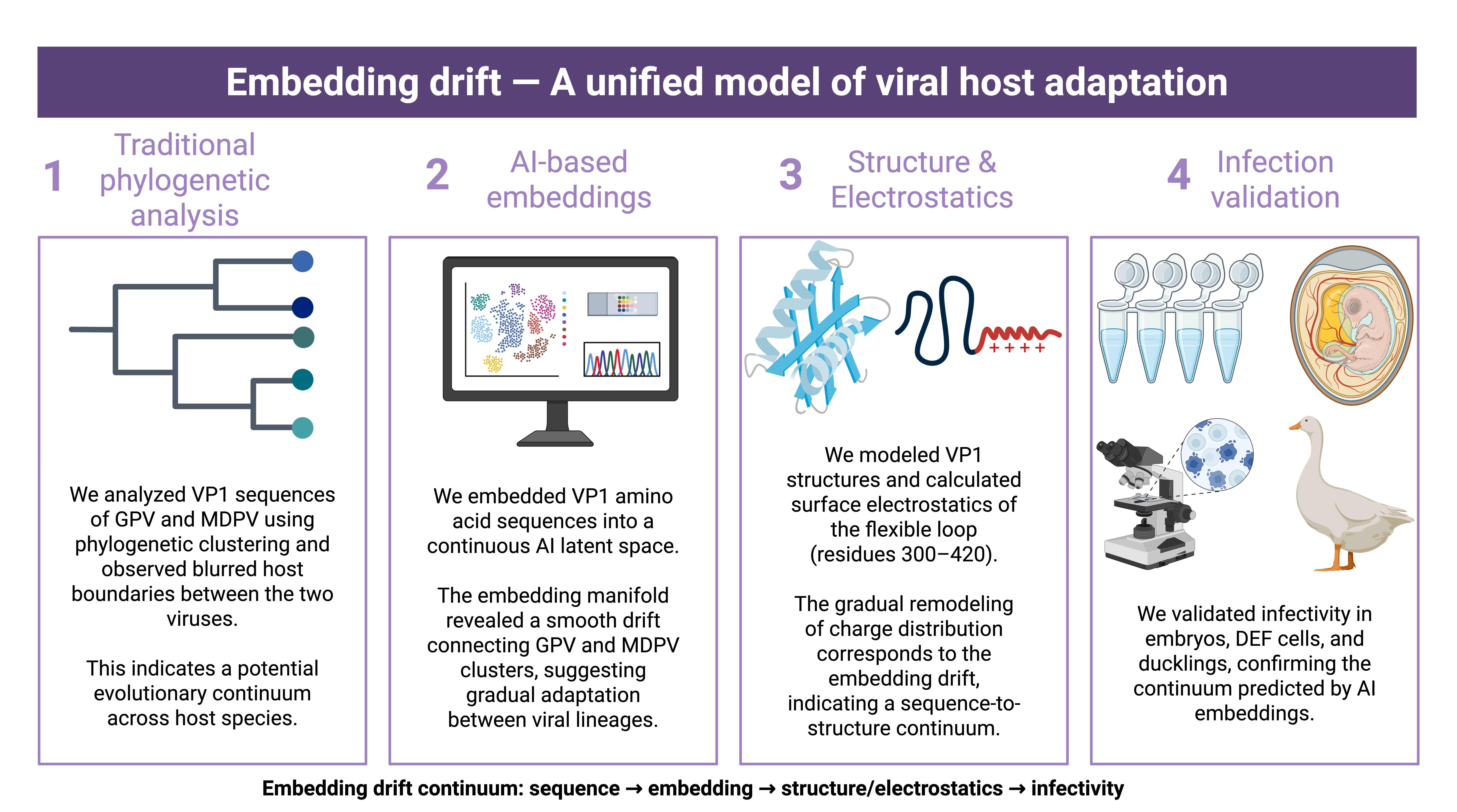

Supplement: Supplementary file 1 [file Image1.png]
